# Risperidone and fluvoxamine; two directions of augmentation: a case report

**DOI:** 10.1192/j.eurpsy.2023.1963

**Published:** 2023-07-19

**Authors:** M. Štracak, I. Todorić Laidlaw

**Affiliations:** 1 Psychiatry, General hospital “Ivo Pedišić”, Sisak, Croatia, Sisak; 2 Psychiatry, Vrapče” Universitiy Psychiatric hospital, Zagreb, Croatia

## Abstract

**Introduction:**

Only 40%–70% of patients have an adequate response to the first-line treatment of OCD with SSRIs. Antipsychotic augmentation is effective in the management of both obsessions and compulsions and these drugs are currently the first-line pharmacological augmenting agents for OCD. (Jaisoorya T, Thamby A. Antipsychotic augmentation in the treatment of obsessive-compulsive disorder. Indian Journal of Psychiatry. 2019;61(7):51)

**Objectives:**

We present the cases of a 31-year-old man and a 21- year-old girl. A 31 -year old man experienced hygienic obsessive and compulsive symptoms with excessive showering for one year (YBCOS 20). First, he was treated with 100 mg of fluvoxamine daily which helped him gain partial remission of symptoms. After a stressful period at work and his showering compulsions got worse. Our female patient suffered from compulsive hand washing and disinfection with fear of getting contaminated by other people (YBCOS 24). She was also treated for anorexia nervosa. At the moment of referral to our outpatient clinic, she had already been taking 2 mg of risperidone daily, but still, her compulsions persisted.

**Methods:**

Both patients were included in outpatient service treatment. They were followed up weekly, psychotherapeutically supported and their psychopharmacotherapy was titrated. In the case of our male patient, risperidone of 1 mg/day was added to the ongoing fluvoxamine of 100 mg/day. Our female patient was given fluvoxamine 50 mg/a day and gradually increased to 100 mg/day with an ongoing 2 mg/day of risperidone.

**Results:**

After two weeks, the hygiene compulsions of the 31-year-old-man completely remitted (YBCOS 6). He stopped excessive showering and became fully functional at work and in family relations. Our female patient also continued to take risperidone in augmentation with fluvoxamine as recommended. Her compulsions improved, and she returned to her hobbies and her college lectures (YBCOS 8). They have both been advised to continue outpatient psychiatric treatment and to regularly use pharmacotherapy.

**Conclusions:**

The condition of both of our patients improved after adding an augmentative agent to the therapy. In the first case, it was risperidone as a fluvoxamine augmentation, and in the second, fluvoxamine was added as a risperidone augmentation. The combination of these two drugs, rather than each other being used on its own, proved to be a powerful therapeutic tool in the treatment of OCD. Further clinical studies are required for a better understanding of the underlying neurobiological mechanism of this effective combination.

**Disclosure of Interest:**

None Declared

